# ‘Self-Protection’ of Individual CD4^+^ T Cells against R5 HIV-1 Infection by the Synthesis of Anti-Viral CCR5 Ligands

**DOI:** 10.1371/journal.pone.0003481

**Published:** 2008-10-22

**Authors:** Yongjun Guan, Sayed Abdelwahab, Roberta Kamin-Lewis, Anthony L. DeVico, George K. Lewis

**Affiliations:** Division of Basic Science and Vaccine Research, Institute of Human Virology, University of Maryland School of Medicine, Baltimore, Maryland, United States of America; University of California San Francisco, United States of America

## Abstract

It is well established that paracrine secretion of anti-viral CCR5 ligands by CD8^+^ and CD4^+^ T cells can block the infection of activated CD4^+^ T cells by R5 and dual-tropic isolates of HIV-1. By contrast, because CD4^+^ T cells can be infected by HIV-1 and at least some subsets secrete anti-viral CCR5 ligands, it is possible that these ligands protect against HIV-1 via autocrine as well as paracrine pathways. Here we use a model primary CD4^+^ T cell response in vitro to show that individual CD4^+^ T cells that secrete anti-viral CCR5 ligands are ‘self-protected’ against infection with R5 but not X4 strains of HIV-1. This protection is selective for CD4^+^ T cells that secrete anti-viral CCR5 ligands in that activated CD4^+^ T cells in the same cultures remain infectable with R5 HIV-1. These data are most consistent with an autocrine pathway of protection in this system and indicate a previously unappreciated selective pressure on the emergence of viral variants and CD4^+^ T cell phenotypes during HIV-1 infection.

## Introduction

It has been known for over a decade that CD8^+^ T cells secrete the anti-viral CCR5 ligands, CCL3 (MIP-1α), CCL4 (MIP-1β), and CCL5 (RANTES), which block the infection of CCR5+ cells by R5 viruses *in vitro* ([1] and reviewed in [2]). Since then, a number of studies have implicated anti-viral CCR5 ligands in protective immunity against HIV-1 in the clinical settings of exposed uninfected cohorts [3], [4], neonatal transmission [5], and progression to AIDS [6]–[9]. Shortly after the original discovery that CD8^+^ T cells synthesize anti-viral CCR5 ligands a study appeared indicating that CD4^+^ T cells also secrete these molecules [10]. Several subsequent studies suggested that synthesis of CCR5 ligands by ex vivo CD4^+^ T cells correlates with resistance of these cells to infection and indicated an inverse relationship between CCR5 ligand synthesis and lower co-receptor levels [3], [11]–[13]. These studies did not place CCR5 ligand synthesis into a precise immunological context defined by the response phase (i.e., primary or secondary) or CD4^+^ T cell subsets synthesizing the ligands.

To this end, our group [14], [15] and another [16], [17] reported the synthesis of a CCR5 ligand, CCL4, by memory CD4^+^ T cell subsets in uninfected volunteers. Interestingly, the frequencies of memory CD4^+^ T cells that synthesize CCL4 in response to antigenic stimulation were substantially lower than those found for CD8^+^ T cells [14], [16]. Collectively, these studies show that CD4^+^ T cells can synthesize anti-viral CCR5 ligands during the memory phase of an adaptive immune response, although CD8^+^ T cell subsets might be the predominant source of these molecules at this point in the adaptive immune response. Less is known about the synthesis of anti-viral CCL5 ligands during the primary CD4^+^ T cell response.

To address this issue, we established an *in vitro* model of the primary antigen specific CD4^+^ T cell response in which purified naïve CD4^+^ T cells from healthy people are co-cultured with monocyte derived dendritic cells (MDDC) plus antigen [18]. The subsequent immune response was measured *in vitro* by activation parameters including the synthesis of anti-viral CCR5 ligands [18]. In this report, we extend those studies by showing that individual CD4^+^ T cells which synthesize anti-viral CCR5 ligands are ‘self-protected’ against infection with R5 but not X4 isolates of HIV-1 during the primary immune response *in vitro*. These data are most consistent with an autocrine mode of protection and indicate previously unappreciated selective pressure on the emergence of viral variants and CD4^+^ T cell phenotypes during HIV-1 infection.

## Materials and Methods

### Reagents

Peripheral blood mononuclear cells (PBMC) were isolated by Ficoll-Hypaque® centrifugation using blood obtained from normal healthy volunteers either through commercial sources or by venipuncture of healthy adult volunteers under approval of the University of Maryland Institutional Review Board or through commercial sources. The following fluorochrome-labeled antibodies were purchased from BD Biosciences (San Jose, CA): CD4-FITC, CD3-PerCP, CD45RO-APC, CD86-PE and CCL4-PE. The antibodies against HIV1 p24 (KC57-RD and KC57-FITC) were obtained from Beckman Coulter (Miami, FL). Neutralizing goat antibodies specific for CCL3, CCL4, and CCL5, along with normal goat IgG, were from R&D Systems (Minneapolis, MN). Staphylococcus enterotoxin B (SEB) was purchased from Sigma (St. Louis, MO).

### Preparation of Virus Stocks

HIV-1 stocks (four R5 viruses: HIV-1_Ba-L_, HIV-1_92BR020_, HIV-1_Jv1083_, HIV-1_SF162_; three R5/X4 viruses: HIV-1_89.6_, HIV-1_92HT594_, and HIV-1_BZ167_; four X4 viruses: HIV-1_92ug024_, HIV-1_Lai_, HIV-1_IIIB_, HIV-1_2044_) were obtained originally from the NIAID AIDS Reference and Research Reagent Repository (Kensington, MD). Working viral stocks were prepared for this study by infection of CD8-depleted PHA-stimulated PBMCs from a single donor using seed stocks maintained in the uQuant Core Facility at the Institute of Human Virology.

### Viral Infection During Primary CD4^+^ T Cell Responses *in vitro*

Generation of immature monocyte-derived dendritic cells (MDDC) and the culture system for generating primary *in vitro* CD4+ T cell responses were described in detail previously [18]. In this system, highly enriched naïve (CD45RO^−^ CD62L^+^) CD4^+^ T cells are cultured with autologous MDDC and nominal antigen, allogeneic MDDC (as alloantigen), or superantigens for periods of up to three weeks. Responses are monitored by cell division by CFSE dilution and for changes in phenotype and function by surface markers and cytokine production, respectively. Responses in this system are strictly dependent upon MDDC as antigen presenting cells and foreign antigen. The ability of this system to detect primary CD4^+^ T cell responses in a clonal fashion has been described [18]. For viral infection, 1000 TCID50 of the indicated HIV-1 isolates were added with naïve CD4+ T cells (2×10^5^ per well), MDDC (2×10^3^ per well) and antigen (100 ng/ml SEB) in 200 µl per well medium in U-bottom 96-well plates. In some experiments, alloantigens were used in lieu of SEB to elicit the primary immune response. This was accomplished by using 1×10^4^ allogeneic MDDC as the stimulus for naïve CD4^+^ T cells. Viral infection of MDDC was performed by co-culturing MDDC (2×10^4^ per well) with 1000 TCID50 of the HIV-1 isolates as indicated in the text. Aliquots of supernatants were removed on days 2, 5, 8, 11, and 14 and quantified for p24 concentrations by ELISA (New England Nuclear, Cambridge, MA). Alternatively, viral replication was determined by intracellular staining for HIV-1 p24 antigen as described below. Each experiment was repeated at least twice with comparable results.

### Intracellular staining

Cells were collected at the time points indicated in the Results section and washed twice in staining buffer [PBS with 2% BSA and 0.1% Sodium azide]. They were stained for surface markers as indicated in the Results section, fixed, and permeabilized for intracellular staining according to manufacturer's instructions using the Cytofix/Cytoperm Plus kit (BD Biosciences, San Jose, CA). For detection of intracellular MIP1β, Golgistop reagent (BD Biosciences, San Jose, CA) was added on day 6 for the last 10 hours of culture before collecting cells for staining as described above. We used the KC57-FITC antibody for detection of intracellular of HIV1 p24 antigen and MIP1β-PE antibody for detection of intracellular CCL4. Experience has shown that it is difficult to reliably detect the two other anti-R5 HIV-1 β-chemokines, CCL3 and CCL5 by intracellular staining. For this reason we were only able to follow the synthesis of CCL4 (MIP-1β). Co-staining for p24 and CCL4 was carried out after the last 10 hours of culture in the presence of Golgistop. Data were acquired by six-parameter flow cytometry and analyzed with FlowJo software (Treestar, Inc., San Carlos, CA).

## Results

### Selective replication of X4 and R5/X4 HIV-1 during the *in vitro* primary CD4^+^ T cell response

**Figure 1:**
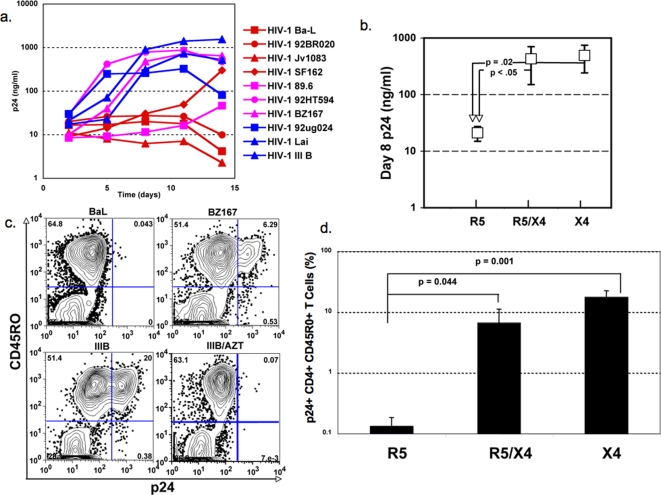
Selective replication of X4 or R5/X4 viruses during the primary *in vitro* CD4+ T cell response to SEB. a. Four R5 viruses (Ba-L, 92BR020, Jv1083, SF162), three R5/X4 viruses (89.6, 92HT594, BZ167) and three X4 viruses (92ug024, Lai, IIIB) were added along with naïve CD4^+^ T cells plus MDDC and SEB to initiate a primary immune response *in vitro* as described in Materials and Methods. The viral input was 1000 TCID50 per culture. Viral replication was monitored by detection of HIV1 p24 antigen in supernatant and shown in red for R5 viruses, magenta for R5/X4 viruses, and blue for X4 viruses. b. Day 8 p24 levels were pooled for each viral co-receptor family and the medians were compared for statistical significance by a double tailed Student's t-test. c. Representative examples are shown of p24 staining for CD4^+^ CD45RO^+^ T cells responding to SEB and autologous MDDC in the presence of a R5 virus (Ba-L), a R5/X4 virus (BZ167), or a X4 virus (IIIB), the latter in the presence and absence of AZT ( 10 uM). Cells were harvested on day 7 post-initiation of a primary CD4^+^ T cell response in the presence of the indicated HIV-1 isolates (1000 TCID50 per culture) and stained for surface markers and p24 antigen as described in Materials and Methods. d. Median frequencies of p24+ CD4^+^ CD45RO^+^ cells were pooled for each co-receptor family and compared for statistical significance by a two-tailed Student's t-test. The data are from staining analyses carried out on day 7 cells generated in a primary CD4^+^ T cell response to SEB in the presence of 1000 TCID50 of the R5, R5/X4, and X4 viruses used in Figure 1a.

The ability of R5, X4, and R5/X4 isolates of HIV-1 to replicate in CD4^+^ T cells during the primary *in vitro* immune response was determined using a system developed by our group to evaluate the synthesis of anti-viral CCR5 ligands [18], the effects of adjuvants on antigen presentation [19]–[21], and the responses to HIV-1 vaccine candidates [22]. Cultures were initiated and infected with the HIV-1 isolates shown in Figure 1a as described in Materials and Methods. As shown in Figure 1a, five of the six X4 and R5/X4 viruses replicated by day 8 of the culture period. The R5/X4 virus HIV-1_89.6_ replicated poorly, if at all. By contrast, none of the four R5 viruses replicated early in the culture period with only HIV-1_SF162_ showing a modest increase in p24 by day 11. Since the differences in replication appeared to be greatest during the first 11 days of culture, we compared the day 8 median p24 concentrations of the three groups for statistical significance using Student's t-test. As shown in Figure 1b, the differences between the R5 group and either the X4 or X4/R5 groups were statistically significant (p = 0.02) for X4 viruses, p≤0.05 for X4/R5 viruses). These data show that X4 and X4/R5 viruses selectively replicate during the antigen driven primary CD4^+^ T cell response *in vitro* with little or no replication of R5 viruses under these experimental conditions. This conclusion was confirmed in subsequent experiments by analysis of intracellular p24.

**Figure 2:**
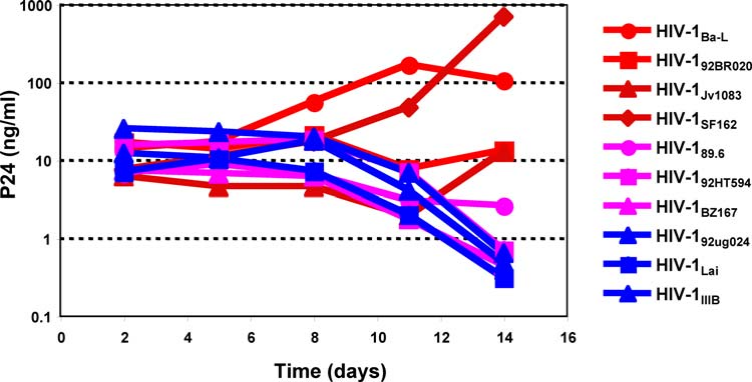
Selective replication of R5 HIV-1 in immature MDDC. Immature MDDC cells ( 2×10^4^ per well) were cultured with 1000 TCID50 of the HIV-1 viruses indicated in the figure panel as described in Methods. Supernatants were collected on days 2, 5, 8, 11 and 14 post-infection and tested for HIV-1 p24 antigen by ELISA. The results are shown in red for R5 viruses, magenta for R5/X4 viruses, and blue for X4 viruses.

The data from one such experiment are shown in Figure 1c and 1d where cells were harvested on day 7 from cultures established as above for Figure 1a washed and stained for intracellular p24 as described in Materials and Methods. Figure 1c shows typical intracellular staining of CD4^+^ CD45RO^+^ T cells for intracellular p24 for cultures infected with an R5 virus (HIV-1_Ba-L_), an R5/X4 virus (HIV-1_BZ167_), or an X4 virus (HIV-1_IIIB_). Note that CD45RO marks responding cells in our assay system[18], [22]. As expected from the results on supernatant p24 (Figure 1a), no p24 staining was found for cultures infected with R5 virus, HIV-1_Ba-L_, whereas p24 staining was apparent for both the R5/X4 and X4 viruses shown in Figure 1c. Staining for p24 was absent when an inhibitory concentration AZT was included in a parallel culture exposed to the X4 virus, showing that the observed staining required active reverse transcription and that the p24 signal was not due to passively adsorbed protein from the input virus. Statistical significance of differential infection as determined by p24 staining was assessed for the same virus panel shown in Figure 1a using the frequencies of CD4^+^ CD45RO^+^ T cells that also express p24. The results of this analysis are shown in Figure 1d where the frequencies of CD4^+^ CD45RO^+^ p24^+^ cells in R5 cultures were approximately 100-fold lower than those observed for the R5/X4 and X4 virus panels. These differences were statistically significant as determined by a two-tailed Student's t-test (p = 0.044 and p = 0.001 for comparisons of the R5 viruses with the R5/X4 and X4 viruses, respectively). These data confirm the conclusions reached using p24 supernatant concentrations as the readout and show that antigen activated CD4^+^ T cells are the probable source of the virus. As the cultures were initiated with both immature MDDC and naïve CD4^+^ T cells, we cannot formally rule out a contribution of the MDDC to the virus found in the supernatants. However, this is unlikely given the small number of MDDC in the cultures (1% at culture initiation) and by additional studies in which R5 viruses selectively replicate in cultures of immature MDDC cultured alone (Figure 2). Thus, it is unlikely that MDDC infection accounts for our results.

### Antibodies to antiviral CCR5 ligands allow the replication of R5 HIV-1 during the primary CD4^+^ T cell response *in vitro*

**Figure 3:**
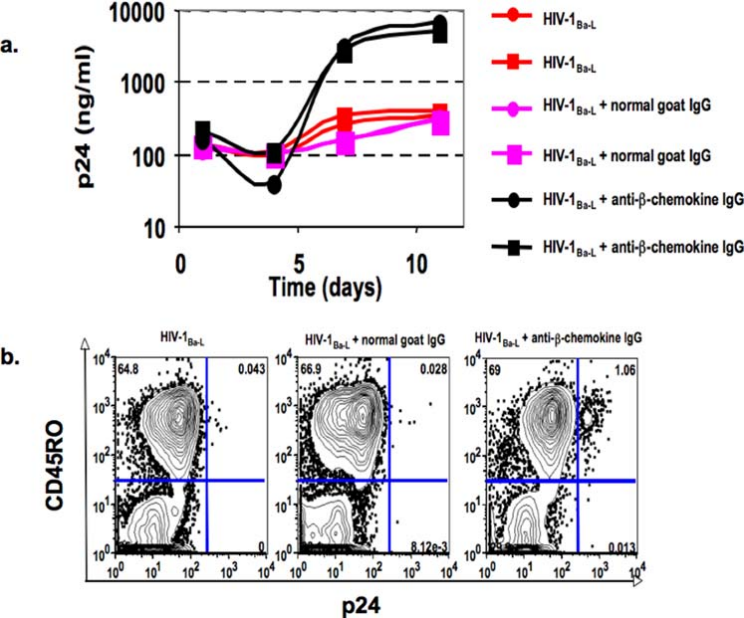
Anti-β-chemokine antibodies reverse the block to R5 HIV-1 replication in the primary *in vitro* response of CD4^+^ T cells to SEB. a. Goat antibodies to CCL3, CCL4, and CCL5 were added at 25 ug/ml (final concentration of each antibody) to cultures of naïve CD4^+^ T cells and autologous MDDC plus SEB in the presence of 1000 TCID50 HIV-1_Ba-L_ and viral replication monitored on the indicated days by p24 ELISA. Controls included cultures initiated in the absence of exogenous antibodies and cultures initiated with normal goat IgG (75 ug/ml final concentration). Supernatant p24 concentrations are shown for duplicate cultures. b. Bivariate histograms for p24 versus CD45RO were generated as described in the legend for Figure 1c to confirm the replication of HIV-1_Ba-L_ in the presence of saturating concentrations of ant-β-chemokine antibodies. The data are shown for a day 7 culture established as described in the legend to Figure 3a.

The poor replication of R5 viruses in the experiments described above strongly suggested that the effect is due to anti-viral CCR5 ligands made by the responding CD4^+^ T cells. To test this hypothesis, cultures were established as described in Figure 1 using HIV-1_Ba-L_ as the R5 virus but spiked with a mixture saturating concentrations of blocking antibodies specific for CCL3, CCL4, and CCL5 or non-immune goat IgG as the negative control. The experimental groups (shown in duplicate in Figure 3a ) include HIV-1_Ba-L_ alone, HIV-1_Ba-L_ with non-immune goat IgG, and HIV-1_Ba-L_ with the mixture of blocking goat anti-β-chemokine IgGs (Fig. 3a, black lines). As expected, no viral replication was observed for groups cultured with HIV-1_Ba-L_ or HIV-1_Ba-L_ plus non-immune goat IgG (Figure 3a, red and magenta lines, respectively). By contrast, viral replication was observed in the group cultured with HIV-1_Ba-L_ plus the mixture of goat anti-β-chemokine IgGs. Intracellular p24 staining confirmed the presence of viral protein synthesis in responding CD4^+^ CD45RO^+^ T cell blasts (Figure 3b) in the cultures treated with the mixture of anti-β-chemokine IgGs (right panel) but not in the cultures treated with non-immune goat IgG (middle panel). These results support the hypothesis that the anti-viral CCR5 ligands, CCL3, CCL4, and CCL5, produced during the primary CD4^+^ T cell response *in vitro* are sufficient to severely dampen replication of an R5 virus present continuously in the culture. They also set the stage in this culture system for determining whether the individual CD4^+^ T cells that secrete anti-viral CCR5 ligands are ‘self-protected’ from infection with R5 HIV-1.

### Individual CD4+ T cells that secrete anti-viral CCR5 ligands during the primary immune response *in vitro* are ‘self-protected’ from concomitant R5 HIV-1 infection

**Figure 4:**
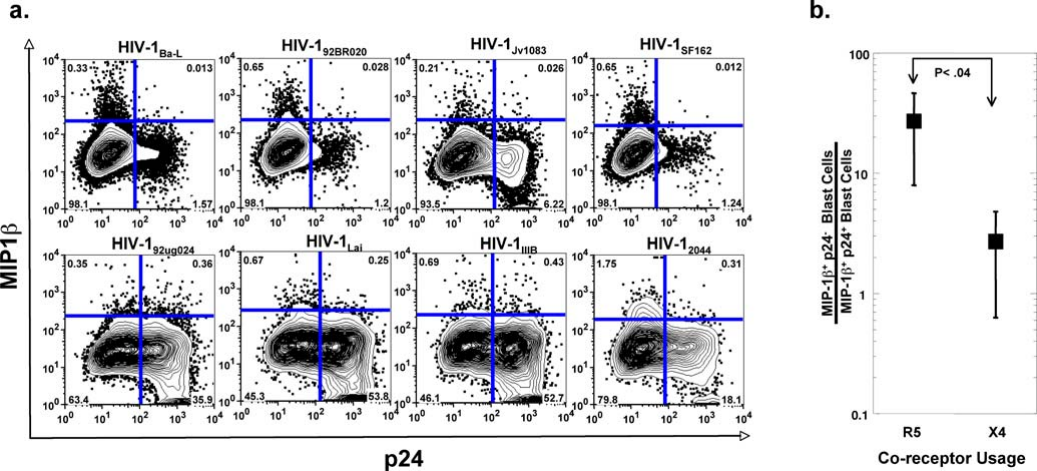
Exclusive expression of p24 and CCL4 in CD4+ T cells undergoing a primary immune response in the presence of R5 but not X4 HIV-1-. a. Naïve CD4^+^ T cells were cultured with allogeneic MDDC in the presence of 1000 TCID50 of the HIV-1 isolates indicated in the figure and intracellular p24 and CCL4 staining was carried out as described in Materials and Methods on cultures harvested at day 7. b. Ratios of CCL4^+^ p24^+^ cells to CCL4^+^ p24^−^ cells were calculated for each virus shown in panel Figure 4a and the medians used to determine statistical significance between the R5 an X4 HIV-1 groups using a two-tailed Student's t-test.

The above results suggest that the individual CD4+ T cells that secrete anti-viral CCR5 ligands might be selectively protected from infection by R5 viruses. This ‘self-protection’ hypothesis predicts that the synthesis of anti-viral CCR5 ligands and viral proteins at the single cell level should tend toward mutual exclusivity for R5 viruses but not for X4 viruses in our culture system. Since we have to add blocking anti-beta chemokine antibodies to establish infections with R5 viruses in the SEB stimulated cultures; it is difficult to test this hypothesis using this potent antigen as the readout. In preliminary studies, we found that using weaker antigenic stimuli, such as allogeneic MDDC, permits modest replication of R5 viruses, which allowed us to test the hypothesis in the absence of blocking anti- β -chemokine antibodies. The study shown in Figure 4 was carried out essentially the same as that shown in Figure 1 except that allogeneic MDDC were used in lieu of SEB and autologous MDDC as antigen to elicit the primary CD4+ T cell response. Single cell analyses were carried out on day 7 of culture by intracellular staining and flow cytometry using fluorescent monoclonal antibodies to CCL4 and p24 as markers for β-chemokine and HIV-1 infection, respectively.

As shown in Figure 4a, there was less apparent coincident staining for CCL4 and p24 in cultures infected with R5 viruses (upper panel) as compared with X4 viruses (lower panel). R5/X4 viruses were not evaluated in this series of studies. The apparent differences in coincident CCL4 and p24 staining were evaluated for statistical significance by calculating the ratios of CCL4^+^ p24^−^ cells to CCL4^+^ p24^+^ cells for the R5 and X4 virus panel and analysis by a two-tailed Student's t-test. As shown in Figure 4b, the ratio of CCL4^+^ p24^−^ cells to CCL4^+^ p24^+^ cells ranged from 8.08 to 54.17 (mean = 27.71±19.24 (SD)) for R5 viruses. By contrast, the ratio of CCL4^+^ p24^−^ cells to CCL4^+^ p24^+^ cells was approximately 10-fold less for X4 viruses, ranging from 0.97 to 5.65 (Figure 4b, mean = 2.73±2.07 (SD)). This difference was statistically significant (p = 0.04) indicating a greater degree of co-incident staining between p24 and CCL4 for the X4 viruses as compared to the R5 viruses. Taken together, these data support the hypothesis that CD4+ T cells which secrete anti-viral CCR5 ligands during the primary immune response *in vitro* are selectively protected from infection by R5 viruses as compared with other activated CD4^+^ T cells in the same culture that do not secrete these ligands. These data are most consistent with an autocrine pathway of protection.

## Discussion

The principal conclusion of this report is that CD4^+^ T cells which secrete anti-viral CCR5 ligands during the primary immune response are ‘self-protected’ from R5 HIV-1 infection. This conclusion is based on kinetic studies of HIV-1 replication during the primary antigen specific CD4^+^ T cells *in vitro* where R5 virus growth was selectively dampened by the synthesis of anti-viral CCR5 ligands by the responding T cells. It is also based on single cell analyses of the CD4^+^ T cells undergoing a primary response where R5 HIV-1 infection and the synthesis of CCL4 were largely exclusive. By contrast, significant exclusivity was not observed for X4 HIV-1 infection. Taken together, these data show that CD4^+^ T cells, which synthesize anti-viral CCR5 ligands during the primary immune response, are “self-protected”. To our knowledge, this is a new phenomenon in the interplay between HIV-1 infection and anti-viral effector mechanisms of CD4^+^ T cells, which are the principal targets of this virus. Four aspects of this observation warrant further discussion.

First, this observation strongly suggests that CD4^+^ T cells manifest potent anti-HIV-1 effector activity early in the primary immune response *in vivo*. It is well established that memory CD8^+^ T cells secrete high levels of anti-viral CCR5 ligands [1], [7], [14] constituting one of several cell-mediated anti-HIV effector mechanisms; however, less is known about the production of these factors by CD4^+^ T cell subsets during the different phases (i.e., primary and secondary) of an immune response. In an early study using CD45RA and CD45RO to mark naïve and memory CD4^+^ T cell subsets, respectively, it was shown that both CD45RA^+^ and CD45RO^+^ subsets activated by anti-CD3 plus anti-CD28 secreted anti-viral CCR5 ligands [23]. Our results showing that antigen-activated naïve CD4^+^ T cells secrete anti-viral CCR5 ligands agree and extend those studies. They agree in that both sets of studies show that recently activated naïve CD4^+^ T cells secrete anti-viral CCR5 ligands. Our studies place this observation on a more solid footing in that the previous study used only CD45RA expression to define naïve CD4^+^ T cells. It is known that a subset of memory CD4^+^ T cells in normal individuals also express CD45RA and that a second marker, such as CD62L needs to be used to exclude this subset from the naïve pool [24]. In the above experiments, naïve cells were defined as CD4^+^ CD45RO^−^ CD62L^+^, which excludes the CD45RA^+^ population of memory CD4^+^ T cells. Because of this, our studies more precisely define the CD4^+^ T cell subsets that secreted anti-viral CCR5 ligands.

Using this marker strategy we showed previously that that CCL4 is made by CD8^+^ and CD4^+^ memory T cells where greater frequencies of CCL4^+^ cells were found for the CD8^+^ subsets [14]. This has been confirmed by others during vaccine evaluation [16]. By contrast, naïve CD4^+^ or CD4^+^ T cells did not synthesize CCL4 in short term assays [14]. However, shortly after immunization *in vitro* with model antigens, naïve CD4^+^ T cells differentiate into blasts that secrete CCL3, CCL4, and CCL5 at levels that are potently anti-viral when culture supernatants are evaluated in *trans* using R5 viruses and assays using CD4^+^ CCR5^+^ indicator cells [18]. This indicated that CD4^+^ T cells could exert potent anti-viral activity early in the primary immune response.

This observation led us to the current studies where we directly challenged antigen-driven primary CD4^+^ T cell responses with R5, R5/X4, and X4 viruses to determine whether these CCR5 ligands are active *in situ*. In the studies described above, the viruses were simply added at the time of culture initiation with antigen, MDDC, and naïve CD4^+^ T cells and viral growth monitored over the course of the primary response. In all cases, strong primary immune responses were observed to the model antigens as judged by high frequencies of CD4^+^ CD45RO^+^ T cells in the stimulated cultures. It should be noted that CD45RO expression is upregulated within hours after antigen stimulation in our culture system ([18] and unpublished). Thus, the presence of HIV-1 during this early phase of the immune response is not suppressive. We used SEB and alloantigens as the immunogens because anti-HIV-1 primary responses are small in magnitude in our system and these responses are highly reproducible among volunteers (unpublished). In addition, the primary alloantigen response provided a fortuitously optimal dynamic range of response for the bivariate analyses of CCL4 synthesis and HIV-1 infection monitored by intracellular CCL4 and p24 expression. This raises one caveat in that our conclusions are based on primary responses to non-HIV-1 antigens, although it is likely that HIV-1 specific T cells are among the spectrum of responding T cells elicited by SEB and alloantigens.

There is another potential caveat in using superantigens as opposed to peptide-MHC complexes (i.e., alloantigens or nominal protein antigens) in that superantigens activated CD4^+^ T cells through both lck-dependent and lck-independent pathways whereas peptide-MHC complexes apparently stimulate only via the former pathway [25], [26]. In this regard, it is important to note that the key observation in the studies described above, ‘self-protection’ of CD4^+^ T cells by the synthesis anti-viral CCR5 ligands, was made using alloantigen (i.e., peptide-MHC) stimulation. This tempers the caveat that the lck-independent pathway contributes to this observation.

Second, the selective pressure exerted by anti-viral CCR5 ligands secreted by CD4^+^ T cells undergoing a primary immune response could affect both the viral and cellular phenotypes that emerge early in HIV infection. Immune responses to different viruses can result in distinct populations of memory T cells that differ not only in the spectrum of their antigen receptor sequences but also surface phenotype and effector function (reviewed in [27]). This is due to the local ecology of the cell types infected by the virus and by adaptation of the responding T cells to effectively confront the virus, which attempts to escape the response by evolution under this selective pressure. In the case of HIV-1, it has been known for some time that distinct T cell subsets emerge during infection [28], [29] and that this is often paralleled by the emergence of viral variants with distinct phenotypes such as altered co-receptor usage [30]–[32], although the two phenomena have not been causally linked by direct experiment. Our studies suggest a mechanism whereby CD4^+^ T cell phenotype can affect viral phenotype and vice versa during a primary immune response.

Since our results show that CD4^+^ T cells secreting anti-viral CCR5 ligands during the primary immune response are selectively protected from R5 but not X4 viruses, HIV-1 specific CD4^+^ T cells with this phenotype should selectively survive during acute infection and contribute ultimately to the memory pool. It is interesting to note that while HIV-1 specific memory CD4^+^ T cells are selectively infected during an ongoing infection as compared with CMV specific memory CD4^+^ T cells, only a fraction of the HIV-1 specific memory cells are infected [33]. While it is possible that this result simply represents the stochastic nature of HIV-1 infection, it is striking that the majority of HIV-1 specific cells were not infected. Our studies suggest that the uninfected HIV-1 specific CD4^+^ T cells might be refractory to infection because they produce anti-viral CCR5 ligands and are ‘self-protected’. If so, we predict that HIV-1 specific memory CD4^+^ T cells that survive during infection secrete anti-viral CCR5 ligands. In this vein, a recent report described the emergence of an unusual CD4^+^ CD45RA^+^ CCR7^−^ CCR5^+^ memory population in infected people that is resistant to HIV-1 infection [34]. It will be interesting to learn whether these memory cells derive from the lineage of CD4^+^ T cells that secrete anti-viral CCR5 ligands during the primary immune response.

Third, it is possible that the synthesis of anti-viral CCR5 ligands early in the primary immune response contributes a selective pressure to generate the latent viral reservoir in resting CD4^+^ T cells [35], [36]. The CD4^+^ T cell reservoir latent harboring HIV-1 is established very early in infection [37], [38] and it persists throughout the course of infection [39], [40] with apparent replenishment even in individuals undergoing successful HAART therapy [39], [40]. Furthermore, this latent pool is populated predominantly by R5 viruses [41]. Viral latency is one means to escape selective pressure and it is tempting to speculate that the selective pressure exerted by anti-viral CCR5 ligands early in the primary immune response contributes to the early establishment of the latent pool of R5 HIV-1. This hypothesis is currently under investigation using our system.

Fourth, our studies also suggest a mechanism that could drive the co-receptor switch early in infection. It is well established that the co-receptor switch can occur rapidly in scid-Hu mice under the selective pressure of CCL5 [42] and it is reasonable to suspect that a similar switch could occur *in vivo* under pressure of CCL5 synthesis by HIV-1 specific CD4^+^ T cells undergoing a primary immune response. We are testing this hypothesis in our system where delayed viral growth is observed for some R5 virus isolates such as HIV-1_SF162_ (Figure 1a). This virus might be particularly susceptible to co-receptor switching as it has been shown that it has a single pathway to X4 usage that is determined by two common mutations in the V3 loop (I309R and A316V) regardless of whether the selection pressure was exerted by culturing in the presence of CCL5 or by switching cellular substrates [42]. Thus, it is possible that the HIV-1_SF162_ that appears late in our culture system is actually co-receptor switched. We are testing this hypothesis but the potent secretion of anti-viral CCL5 ligands in the early primary CD4^+^ T cell response *in vitro* [18] coupled with the selective protection of the CD4^+^ T cells that synthesize these ligands, strongly suggests a role for anti-viral chemokines in protection against R5 viruses early in infection.

In summary, the results described above indicate a previously overlooked aspect of protective immunity against HIV-1, the selective protection of CD4^+^ T cells that secrete anti-viral CCR5 ligands during the primary immune response. At this point, our studies are limited to a model system in which naïve CD4^+^ T cells are co-cultured with MDDC and an antigen to elicit a primary immune response. Our studies suggest that HIV specific CD4^+^ T cells that secrete anti-viral CCL5 ligands should be selectively protected during the primary HIV-1 infection and enter the memory pool. It is also possible that the relative survival of these cells is a determining factor in the relationship between the magnitude of early CD4^+^ T cell depletion and clinical outcome in SHIV vaccination models [43]. Most important, these conclusions suggest that the design immunogens which selectively elicit CD4^+^ T cell responses capable of “self-protection” against HIV-1 is a potential new strategy in the quest for an AIDS vaccine.
